# The prevalence and determinants of impaired fasting glucose in the population of Taiwan

**DOI:** 10.1186/1471-2458-13-1123

**Published:** 2013-12-05

**Authors:** Chen-Mei Chen, Mei Chang Yeh

**Affiliations:** 1 Department of Nursing, Chang Gung University of Science and Technology, Tao-Yuan, Taiwan; 2 Department of Nursing, College of Medicine, National Taiwan University, Taipei, Taiwan

**Keywords:** Impaired fasting glucose (IFG), Prevalence, Adult, Epidemiology, Taiwan

## Abstract

**Background:**

A current prevalence and relevant risk factors for impaired fasting glucose (IFG) have been reported by various ethnic groups and countries. By contrast, nationwide data for the incidence of IFG in Taiwan have not been presented in the past 15 years. The aim for this manuscript was to estimate the prevalence of IFG and associated risk factors in the population of Taiwan.

**Methods:**

For this cross-sectional research, we used a nationally representative sample (*N* = 2604) obtained from the 2005–2008 Nutrition and Health Survey in Taiwan (NAHSIT), and adopted a stratified multistage sampling design. The tools employed for data collection included questionnaire interviews, anthropometry measurements, and laboratory analysis.

**Results:**

The prevalence of IFG among adults in Taiwan is 35.8% (a fasting glucose level between 100 and 125 mg/dl is considered abnormal). An estimated number of people with IFG is 6.5 million. A higher prevalence of IFG is observed in men younger than 65 years compared to women. However, this trend is reversed for the elderly population. The factors significantly associated with IFG include the following: sex, age, overweight (27 > BMI ≥ 24), obesity (BMI ≥ 27), waist circumference (men ≥ 90 cm, women ≥ 80 cm), hypertension, and hyperlipidemia.

**Conclusions:**

IFG among adults in Taiwan is a health concern that requires attention. We recommend targeting the younger population, especially overweight and obese men between the ages of 19 and 40 years, to provide applicable healthy lifestyle counseling and services. Furthermore, appropriate screening of elderly people is required to detect undiagnosed IFG cases and provide early intervention and treatment.

## Background

Diabetes has become increasingly prevalent. According to the International Diabetes Federation (IDF), approximately 366 million adults were diagnosed with diabetes in 2011, and 552 million adults are expected to be affected by diabetes by 2030. In other words, the prevalence of diabetes is predicted to increase by 50.7% over a 19-y period [1]. A survey conducted by Taiwanese researchers from 1993 to 1996 showed that the prevalence of diabetes among people aged over 19 years was 5.3%. This rate increased to 9.1% between 2005 and 2008, nearly double that for 1993 to 1996 [2]. Thus, diabetes has become a critical public health topic that demands global attention. Furthermore, diabetes is associated with greater risks of capillary and macrovascular complications and premature death [3]. Consequently, early screening to identify people at high risk of diabetes is crucial for diabetes prevention.

Impaired fasting glucose (IFG) is a common glucose disorder, and considered a state of prediabetes associated with increased risk of diabetes [4]. IFG refers to a condition in which fasting blood glucose is elevated above normal levels, although the abnormality has not reached the threshold for a diagnosis of diabetes. Recently, IFG has received increasingly widespread attention and is considered a potentially crucial indicator for preventing diabetes and cardiovascular diseases [5]. In 1997, the American Diabetes Association (ADA) defined the threshold for IFG as between 110 and 125 mg/dl [6]. In addition to a substantially higher risk of type 2 diabetes [7,8], a long-term state of abnormal blood glucose increases the risk of cardiovascular disease, stroke, and metabolic syndrome [7]. Therefore, in 2003, the ADA recommended that the threshold for a diagnosis of IFG be reduced from the previous 110 mg/dl to 100 mg/dl [4,9]. This was because people with a fasting blood glucose level of between 100 and 109 exhibit a higher prevalence of diabetes compared to people with a fasting blood glucose level of <100 mg/dl. In addition, fasting blood glucose levels are commonly used as an indicator in clinical tests [10]. Thus, reducing the IFG threshold can facilitate the early identification of people at high risk of diabetes and the adoption of preventive measures that delay the development of type 2 diabetes.

Contrary to the relative irreversibility of diabetes, IFG can be treated using appropriate intervention measures, thereby delaying or preventing diabetes [11]. Nonetheless, because IFG does not typically present with clinical symptoms, confirmation and interventions should begin at an earlier stage. A current prevalence and relevant risk factors for IFG have been reported by various ethnic groups and countries [12-18]. By contrast, nationwide data for the incidence of IFG in Taiwan have not been presented in the past 15 years [7,19]. Therefore, this research adopted the new IFG diagnostic threshold (100–125 mg/dl) to analyze data obtained from the nationwide Nutrition and Health Survey in Taiwan (NAHSIT) for 2005 to 2008. The research objectives were to understand the prevalence of IFG among adults in Taiwan, and to explore the crucial variables that influence Taiwanese adults’ IFG levels. The results of an in-depth investigation of IFG prevalence in Taiwan and other relevant factors can be compared with international research to identify the potential causes of IFG and provide a reference for the planning of future diabetes interventions.

## Methods

We analyzed data from the NAHSIT, which adopted a multistage, stratified clustering sampling scheme [2,20]. The NAHSIT is a national field survey conducted by Academia Sinica under the commission of the Ministry of Health and Welfare, Executive Yuan, Taiwan. The NAHSIT targets children aged 0 to 6 years and adults aged over 19 years who have Taiwanese citizenship, excluding residents of long-term care institutions. The questionnaire response rate was 65%, and the physical examination rate was 59% [20-22]. The study design was previously employed elsewhere [2,20]. The survey implementation and instruments were approved by the Institutional Review Board of Academia Sinica, which also conducted the survey, and reviewers from the Department of Health, Taiwan (DOH94-FS-6–4). The NAHSIT is a cross-sectional and nationally representative survey that was conducted from January 1, 2005, to December 31, 2008.

Briefly, 358 townships and city districts in Taiwan were divided into 8 sampling strata according to urbanization, dietary patterns, and geographic location. These strata comprised 2 in Northern Taiwan and one stratum each for Central Taiwan, Southern Taiwan, Eastern Taiwan, Hakka and mountainous areas, East Coast, and Peng-Hu Islands. Using probabilities proportional to size, sex and townships were selected in each stratum. Overall, 48 townships were chosen for the 8 strata, and 128 individuals were sampled from the selected townships. An eligible person’s probability of selection was based on his/her registered area of residence, sex, and age. Appropriate weights for each stratum were estimated based on the total population in the stratum. This weighting was used in all analyses of the participants’ characteristics included in the questionnaire interview and health examination.

The total NAHSIT sample numbered 6144, comprising 1479 children and 4665 adults aged over 19 years. Among the adult participants, 2808 underwent a physical examination and completed the survey questionnaire. This research analyzed only data of these 2808 participants. After adjusting for age, no major differences in the distribution for sex and education level were observed between the participants who had/had not completed the questionnaire and the participants who had/had not undergone a physical examination [20]. After excluding those who lacked FPG values (*N* = 124) and provided incomplete data (*N* = 80), 2604 participants (1285 men and 1319 women) aged between 19 and 98 years remained.

### Investigated measurements

The tools for data collection included questionnaire interviews, anthropometric measurements, and laboratory analysis.

### Questionnaire data

The researchers administered a structured questionnaire regarding demographic characteristics, namely, age, sex, medical history, and drug consumption, to each participant.

### Anthropometric data

The participants’ body mass index (BMI) was calculated as weight (kg) divided by height squared (m). Waist circumference was measured at the narrowest point between the lower borders of the rib cage and iliac crest. Blood pressure (BP) was measured using an Omega 1400 automatic blood pressure monitor. The systolic blood pressure (SBP) and diastolic blood pressure (DBP) of each participant were measured 3 times using a mercury sphygmomanometer, with the participants in a sitting position, and after 15-min rest periods; the three values were then averaged. Using BMI (as defined by the Department of Health, Taiwan) as the standard for determining obesity, a BMI of 24 to 27 was regarded as overweight and a BMI ≥ 27 as obese [23]. In addition to BMI, waist circumference was sued to indicate abdominal obesity. The standard threshold for this indicator was defined as a circumference ≥ 90 cm for men and ≥ 80 cm for women [24].

### Laboratory data

A fasting blood sample was obtained in the morning after at least an 8-h fast. Laboratory data included levels of fasting blood glucose, total cholesterol (TCHO), triglycerides (TG), high-density lipoprotein (HDL), and low-density lipoprotein (LDL).

### Definition of IFG, hypertension, and hyperlipidemia

Regarding the definition of IFG adopted for this paper, we referenced the 2003 ADA-revised threshold for normal FPG, abnormal FPG (ie, IFG or pre-diabetes), and diabetes [4,9]. Normal FPG refers to an FPG level below 100 mg/dl, without a history of diabetic medication. IFG refers to a level between 100 and 125 mg/dl with no consumption of diabetic medication. Diabetes is manifest when the FPG level equals or exceeds 126 mg/dl, or a history of diabetic medication exists. In addition, we defined hypertension according to the most recent criteria established by the Joint National Committee/World Health Organization (JNC7/WHO) in 2003; specifically, a diagnosis of hypertension is confirmed when a patient’s SBP equals or exceeds 140 mmHg, and/or the patient’s DBP equals or exceeds 90 mmHg [25]. The participants who reported a history of hypertension or current consumption of antihypertensive medication during the survey were classified as having hypertension. We defined hyperlipidemia based on the classification standard developed by the Department of Health, Executive Yuan, Taiwan (2007), which classifies dyslipidemia into the following three types: (1) hypercholesterolemia, if TCHO  ≥ 200 mg/dl; (2) mixed hyperlipidemia, if TCHO  ≥ 200 mg/dl and TG  ≥ 200 mg/dl; and (3) hypertriglyceridemia, if TG  ≥ 200 mg/dl and high-density lipoprotein cholesterol (HDL-C) < 40 mg/dl or TCHO/HDL-C  ≥ 5. Hyperlipidemia is manifest if any of the aforementioned conditions are observed [26]. The participants who reported a history of hyperlipidemia or current consumption of hypolipidemic medication during the survey were classified as having hyperlipidemia.

### Statistical analysis

SPSS (Version 12.0; SPSS Inc., Chicago, Illinois, U.S.A.) was used to conduct data analysis. For descriptive statistics, categorical variables were described using frequency distribution and percentage, and continuous variables were described using the mean and standard deviation (*SD*). For correlational statistics, we conducted an independent samples *t*-test and a chi-square test. For inferential statistics, logistic regression was performed to examine IFG-related factors. The strength of association between the dependent variables was assessed using odds ratios (ORs). Finally, for multiple logistic regression analysis using the backward stepwise conditional method, we included variables found to have a statistically significant association with the dependent variable. All tests conducted in this research were two-tailed tests, and the level of statistical correlation was set as α=0.05.

## Results

### Prevalence of IFG

**Figure 1:**
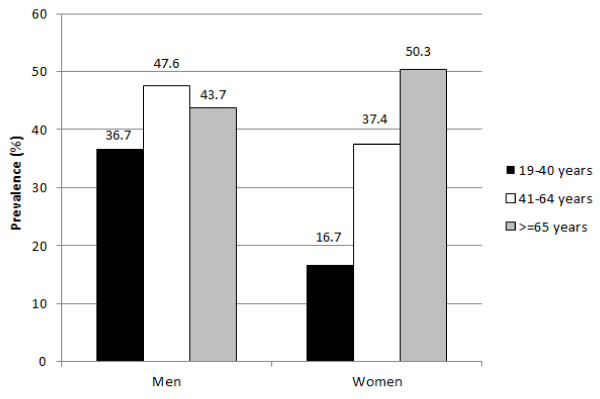
Prevalence of impaired fasting glucose by sex according to age group.

A total of 2604 people participated in this research (1285 men and 1319 women). The participants were aged between 19 and 98 years (mean = 53.8 ± 17.4). According to the 2003 ADA-revised IFG diagnostic threshold (100–125 mg/dl), the prevalence of normal FPG among the participants was 55.7%, and that of IFG was 35.8%. An estimated number of people with IFG is 6.5 million. Overall, the prevalence of IFG was higher among men (42.1%) than among women (29.6%), as shown in Table 1. Furthermore, classifications for the 3 age groups according to sex show that the prevalence of IFG ranged from 36.7% to 43.7% among young (19 to 40 years), middle-aged (41 to 64 years), and elderly (age ≥ 65 years) men. By contrast, the prevalence of IFG in women increased with age and continued to rise among elderly women, reaching 50.3% (Figure 1).

**Table 1:** Estimated prevalence of impaired fasting glucose in Taiwan, 2005–2008

	**Total (N = 2604)**	**Weighted frequency (%)**		

		**Normal**	**IFG**	**DM**
1997 ADA definition as FPG 110-125 mg/dl	18435969	14931484(81.0)	1937615(10.5)	1566870(8.5)
2003 ADA definition as FPG 100-125 mg/dl	18435969	10269578(55.7)	6599521(35.8)	1566870(8.5)
Sex				
Men (n = 1285)	9197400	4392250(47.8)	3868773(42.1)	936377(10.1)
Women (n = 1319)	9238569	5877328(63.6)	2730748(29.6)	630493(6.8)

Abbreviation: *IFG* impaired fasting glucose; *DM* diabetes; *ADA* American Diabetes Association; *FPG* fasting plasma glucose.

Weighted frequency (based on sample weights).

### Associated factors

Compared to those with normal FPG levels, the participants with IFG tended to be older and had relatively higher TCHO, TG, and LDL levels; greater SBP, DBP, BMI, and waist circumference; and yet showed comparatively lower HDL levels. These data demonstrate the statistically significant differences (*P* < 0.01) observed. Overall, the overweight, obesity, abdominal obesity, hypertension, and hyperlipidemia values for the participants with IFG were significantly higher than those for the participants with normal FPG levels (*P* < 0.01; Table 2).

**Table 2:** Comparison of the levels of age, lipids, BP, BMI and WC between IFG and normal participants

**Variables**	**Overall**	
	**Normal**	**IFG**
Age	38.7±14.4	48.2±15.4^*^
Total cholesterol (mg/dl)	184.3±34.3	194.2±37.6^*^
Triglycerides (mg/dl)	98.9±61.4	138.7±111.0^*^
LDL (mg/dl)	115.6±32.4	124.0±36.2^*^
HDL (mg/dl)	57.2±14.6	51.2±13.9^*^
Systolic blood pressure (mmHg)	106.6±13.1	116.5±17.1^*^
Diastolic blood pressure (mmHg)	66.8±10.2	72.5±11.3^*^
Body mass index (Kg/m^2^)	22.5±3.4	25.0±3.7^*^
Waist circumference (cm)	76.4±9.5	84.3±9.9^*^
Men ≥ 90 cm	80.1±8.9	86.7±9.0^*^
Women ≥ 80 cm	73.7±8.9	80.9±10.2^*^
Overweight (%)	21.4	31^*^
Obesity (%)	9.1	27. 8^*^
Abdominal obesity (%)	17.8	43.2^*^
Hypertension (%)	7.5	23.7^*^
Hyperlipidemia (%)	38.5	57.4^*^

Data are means ± *SD* or n (%). ^*^P < 0.01.

### OR of the factors associated with IFG

As shown in Table 3, the logistic regression analysis results indicated that the following variables significantly influence IFG: sex, age, overweight (27 > BMI ≥ 24), obesity (BMI ≥ 27), abdominal obesity (ie, waist circumference; men ≥ 90 cm and women ≥ 80 cm), hypertension, and hyperlipidemia. The likelihood of IFG for men exceeded that for women by 1.99 (95% CI = 1.99–2.00), increasing by 1.03 for each additional year of age (95% CI = 1.03–1.04). Classifications of BMI and waist circumference based on the diagnostic criteria for metabolic syndrome [20,21] indicate that compared to the participants who were not overweight, obese, or categorized as abdominally obese, the participants who were overweight (27 > BMI ≥ 24), obese (BMI ≥ 27), or categorized as abdominally obese according to their waist circumference measurements (men ≥ 90 cm and women ≥ 80 cm) exhibited a 1.44 (95% CI = 1.43–1.44), 2.74 (95% CI = 2.73–2.75), and 1.63 (95% CI = 1.63–1.64) higher probability of IFG, respectively. Additionally, the likelihood of IFG was 1.42 (95% CI = 1.42–1.43) and 1.17 (95% CI = 1.16–1.17) greater for participants with hypertension or hyperlipidemia than for participants without such conditions. In addition, separate analyses of the data for men and women show that crucial shared variables influenced IFG values.

**Table 3:** ORs and 95%CIs for IFG compared with normal glucose from multiple logistic regression model

**Variables**	**Overall**	**Men**	**Women**
	**OR (95%)**	**OR (95%)**	**OR (95%)**
Sex			
Women	1.00 (Reference)		
Men	1.99^*^(1.99-2.00)		
Age	1.03^*^(1.03-1.04)	1.03^*^(1.03-1.03)	1.04^*^(1.04-1.04)
BMI group			
BMI < 24	1.00	1.00	1.00
27 > BMI ≥ 24 (Overweight)	1.44^*^(1.43-1.44)	1.38^*^(1.38-1.39)	1.69^*^(1.68-1.70)
BMI ≥ 27 (Obesity)	2.74^*^(2.73-2.76)	4.01^*^(3.98-4.03)	2.16^*^(2.15-2.17)
Abdominal obesity			
No	1.00	1.00	1.00
Yes	1.63^*^(1.63-1. 64)	1.54^*^(1.53-1.55)	1.46^*^(1.45-1.46)
Hypertension			
No	1.00	1.00	1.00
Yes	1.42^*^(1.42-1.43)	1.33^*^(1.32-1.33)	1.53^*^(1.52-1.54)
Hyperlipidemia			
No	1.00	1.00	1.00
Yes	1.17^*^(1.16-1.17)	1.06^*^(1.05-1.06)	1.25^*^(1.25-1.26)

^*^P < 0.01.

Logistic regression: associated risk factors were selected stepwise in model taking IFG as a dependent variable. Variables included in the model were: sex, age, BMI group, abdominal obesity, hypertension, and hyperlipidemia.

## Discussion

The research participants were Taiwanese adults aged over 19 years. For this investigation, we adopted a cross-sectional research design and stratified multistage cluster sampling method. Thus, the results are probably sufficiently representative of the status of IFG among adults in Taiwan. The first objective of this research was to understand the prevalence of IFG among adults in Taiwan. The findings indicate a prevalence rate of 35.8% (according to the ADA-revised diagnostic threshold for IFG, ie, 100–125 mg/dl). As shown in Table 1, the prevalence of IFG in Taiwan exceeded that in South Korea (23.9%) [13], and was similar to that observed in Wuxi City, China (33.3%). These findings were based on the ADA-revised diagnostic threshold [18]. If the previous threshold for a diagnosis of IFG (ie, 110–125 mg/dl) is used for calculation, the prevalence of IFG in Taiwan is 10.5% (Table 1), which is similar or even lower than that of other countries (eg, 10.0% in the United States, 11.8% in Denmark, and 17.3% in Sweden) calculated using the previous diagnostic threshold [5,27]. In this investigation, we used the 2005–2008 NAHSIT to conduct data analysis. The prevalence of IFG in the aforementioned nations was investigated for years 2001 to 2004. Only the investigation of IFG prevalence in Wuxi City, China, examined data for 2007, which was the same year analyzed in this research. The factors that may contribute to the disparate prevalence of IFG in various countries include era, race, and region. Among the previous studies of IFG prevalence conducted on various workplaces and regions in Taiwan [7,19], one study investigated a large-scale database comprising physical examination data of civil servants from 1989 to 1992, and calculated an IFG prevalence of 27.7% for that period [7], which is considerably lower than the rate determined in this investigation.

In this research, we investigated the distribution of IFG prevalence according to sex and found that the prevalence of IFG among men exceeded that among women. A similar phenomenon was observed abroad [12,13,16]. In addition, the trend of diabetes prevalence in Taiwan shows variations according to sex; that is, the incidence of diabetes was higher among men than among women [2]. Variations according to age were also observed. Specifically, below 65 years of age, men exhibited a higher prevalence of IFG compared to women. We also found that below 65 years of age, men exhibited a higher overweight/obesity prevalence compared with women, although the prevalence among women increased with age and showed a continuous rise among elderly women. IFG is significantly correlated with overweight and obesity. Over the age of 65, IFG prevalence among women was higher than that among men. This is presumably because women have a comparatively longer lifespan, and may also be influenced by the effects of menopause. Furthermore, a drastic increase in IFG prevalence was observed among men aged between 19 and 40 years, which is a warning sign that requires substantial attention. For young men, long-term exposure to IFG risks can lead to the early emergence or development of diabetes-induced pathological capillary changes.

The second research objective was to explore the crucial variables that influence the prevalence of IFG among adults in Taiwan. The results of multiple logistic regression analysis show that the following variables are considerably associated with IFG: sex, age, overweight (27 > BMI ≥ 24), obesity (BMI ≥ 27), abdominal obesity (ie, a waist circumference of 90 cm for men ≥ and ≥ 80 cm for women), hypertension, and hyperlipidemia. This finding is similar to that reported in previous literature [12,16,18,19]. People with IFG have higher cardiovascular risks than people with normal FPG. In addition, clinical research has shown that IFG leads to a collection of symptoms (eg, metabolic syndrome) and is considerably associated with the risk of diabetes [28,29]. The results of this research support those of previous studies conducted in various countries with different ethnic groups by identifying similar factors that correlate significantly with IFG. This further confirms that IFG is frequently comorbid with the risk factors for cardiovascular disease, such as hypertension and dyslipidemia.

In addition, we found that IFG is considerably associated with overweight (27 > BMI ≥ 24), obesity (BMI ≥ 27), and abdominal obesity. Considering that IFG is typically comorbid with the risk factors for cardiovascular disease, including hypertension and dyslipidemia, and highly associated with a 10-y diabetes risk, obesity can further increase these risks [19,30]. Obesity has become increasingly prevalent in Taiwan because of economic growth and lifestyle changes. The following findings were based on the results of 2 domestic nutrition and health surveys conducted from 1993 to 1996 and from 2005 to 2008: The average waist circumference increased by 5.1 cm for adult men and by 4 cm for adult women, and the prevalence of overweight/obesity increased by 17.6% for adult men and by 2.4% for adult women. This shows that the prevalence of overweight and obesity has increased for both men and women in Taiwan, although a greater increase is observed for men [31]. Consequently, active efforts are necessary to develop health promotion programs for this specific population group, using lifestyle changes as the primary treatment method for preventing or delaying diabetes.

Although IFG typically manifests without clinical symptoms, the associated risk of type 2 diabetes is relatively high, and the risk of cardiovascular diseases is increased. Numerous previous studies investigating the risk factors and epidemiology of diabetes have reported that by the time diabetes is diagnosed, the person’s pancreatic β cells typically exhibit a 50% decline in function [32]. Furthermore, diabetes is a chronic disease of slow progression. The risks of cardiovascular disease and stroke are increased if blood glucose levels are slightly higher than normal, yet below the diagnostic threshold for diabetes [7]. However, IFG is a clinical condition and considered reversible [33]. Research regarding IFG-related factors is crucial for initiating prevention and treatment efforts during the early preventive stage, and for encouraging the provision of adequate attention and early interventions to effectively prevent or delay the development of diabetes and relevant complications.

Several limitations of this research must be addressed. First, this research sampled only adults aged ≥ 19 years; thus, the results cannot be extrapolated to other age groups. Second, although the results are based on a random sampling of national data, they only apply to the population of Taiwan. Finally, the data collected using the cross-sectional survey was only for the specific period the survey was conducted, and cannot be used for predictions or as evidence of causality.

## Conclusion

IFG among adults in Taiwan is a health concern that requires substantial attention. Current treatment and prevention methods primarily focus on identifying, preventing, and controlling various risk factors. Therefore, we recommend the following strategies: (1) Target relatively younger population groups, especially men aged between 19 and 40 years who are overweight and obese, with the provision of appropriate healthy lifestyle counseling and services. (2) Provide suitable screening for elderly people to identify undiagnosed IFG cases and provide early intervention and treatment. Thus, the goals of preventing the high-risk group from developing diabetes, and preventing the low-risk group from becoming high risk can be achieved. (3) Future longitudinal follow-up studies should be conducted to provide insights into the causes and effects of IFG, thereby facilitating a more accurate understanding and superior control of IFG incidence in Taiwan. Furthermore, the results of such research can provide beneficial data as a reference for public health-related decisions.

## Abbreviations

ADA: American diabetes association; BMI: Body mass index; CI: Confidence interval; DBP: Diastolic blood pressure; DM: Diabetes; FPG: Fasting plasma glucose; HDL: High-density lipoprotein; IDF: International diabetes federation; IFG: Impaired fasting glucose; IGT: Impaired glucose tolerance; LDL: Low-density lipoprotein; NAHSIT: Nutrition and health survey in Taiwan; NFG: Normal fasting glucose; OR: Odds ratio; SBP: Systolic blood pressure; TCHO: Total cholesterol; TG: Triglycerides.

## Competing interests

The authors declare that they have no competing interests.

## Authors’ contributions

CC researched data, wrote/reviewed the manuscript, and contributed to the conclusion. MY contributed to the conclusion and edited the manuscript. MY is the guarantor of this research and, as such, had full access to all the data and takes responsibility for the integrity of the data and the accuracy of the data analysis. Both authors have read and approved the final manuscript.

## Pre-publication history

The pre-publication history for this paper can be accessed here:

http://www.biomedcentral.com/1471-2458/13/1123/prepub
